# The safety and effectiveness of volumetric magnetic resonance-guided high-intensity focused ultrasound treatment of symptomatic uterine fibroids: early clinical experience in China

**DOI:** 10.1186/s40349-016-0072-9

**Published:** 2016-11-03

**Authors:** Rui Chen, Bilgin Keserci, Hui Bi, Xiaobing Han, Xiaoying Wang, Wenpei Bai, Yueling Wang, Xuedong Yang, Jian Yang, Juan Wei, Minna Seppälä, Antti Viitala, Qinping Liao

**Affiliations:** 1 Department of Obstetrics & Gynecology, Beijing Tsinghua Changgung Hospital, Medical Center, Tsinghua University, No. 168 Litang Road, Changping District, Beijing, China; 2 MR Therapy Division, Philips, Seoul, South Korea; 3 Department of Obstetrics & Gynecology, Peking University First Hospital, Beijing, China; 4 Department of Obstetrics & Gynecology, First Affiliated Hospital of Xi’an Jiaotong University, Xi’an, China; 5 Department of Radiology, Peking University First Hospital, Beijing, China; 6 Department of Radiology, The First Affiliated Hospital of Xi’an Jiaotong University, Xi’an, China; 7 Philips Research China, Shanghai, China; 8 MR Therapy Division, Philips, Helsinki, Finland

**Keywords:** MR-guided high-intensity focused ultrasound, Therapy outcome, Uterine fibroids, Volumetric ablation technique

## Abstract

**Background:**

Uterine fibroids are the most common benign tumor in women, and surgical intervention is still the main fibroid treatment. Patient demands have encouraged development of less-invasive methods such as high-intensity focused ultrasound (HIFU). This study aimed to evaluate the safety and effectiveness of magnetic resonance-guided high-intensity focused ultrasound therapy using a volumetric ablation technique in the treatment of symptomatic uterine fibroids in China.

**Methods:**

One hundred and seven patients were enrolled and treated with magnetic resonance-guided high-intensity focused ultrasound in this study. Clinical efficacy was based on the proportion of patients with fibroid shrinkage (10 % volume reduction or more compared to baseline) at 6 months post treatment as measured with magnetic resonance imaging. The quality of life and symptom outcome was assessed using the uterine fibroid symptom and quality of life questionnaire with symptom severity scoring. Safety was primarily assessed by evaluating the reported adverse events.

**Results:**

Ninety nine of the 107 treated patients had fibroid shrinkage at 6 months post treatment. Resulting in an overall 93 % (95 % confidence interval 86–97 %) treatment success rate, *p* value <0.001; the symptom severity scoring and health-related quality of life at 6 months was statistically different from the screening symptom severity scoring at 0.05 level. Of 366 adverse events reported, there were no study procedure-related or device-related serious adverse events were in the study.

**Conclusions:**

This study demonstrated that the volumetric magnetic resonance-guided high-intensity focused ultrasound device is safe and technically effective and can be utilized in clinically efficient treatments of symptomatic uterine fibroids.

**Trial registration:**

NCT01588899

## Background

Uterine fibroids are the most common benign tumor in pre- and peri-menopausal women. As fibroids increase in size, they can produce pain, menorrhagia, fertility problems, pressure, bloating, and urinary and bowel compression symptoms [1–4].

Treatment options for symptomatic uterine fibroids include surgery (myomectomy or hysterectomy), drug treatment (gonadotropin-releasing hormone analogs) [5], minimally invasive techniques such as uterine artery embolization (UAE) [6–8], and cryotherapy [9, 10]. Even though surgical interventions of uterine fibroids have been the most common treatment option, it requires anesthesia, hospital stays, and long recovery periods. It has been estimated that 600,000 hysterectomies are performed per year in the USA and more than half of the conducted hysterectomies are due to fibroids [11, 12]. Recently, patient demands have encouraged development of less-invasive methods such as UAE, ultrasound-guided high-intensity focused ultrasound (US-HIFU) and magnetic resonance-guided high-intensity focused ultrasound (MR-HIFU).

In HIFU treatment, the beam of HIFU penetrates through soft tissue creating localized high temperatures (55 to 70 °C) for a few seconds within the target, thus producing irreversible cell damage and coagulative necrosis. Applying HIFU energy to a fibroid tissue requires treatment planning, targeting of the US beam to the desired locations and monitoring of the energy delivery. In some applications, this can be performed using diagnostic US imaging in combination with the HIFU. While diagnostic US provides some anatomical details, helps with treatment planning and targeting, and has a strength in terms of motion, for instance, fibroid motion caused by respiration or motion of adjacent bowel that can cause artifact in MR imaging, it does not provide 3D planning or means of measuring the temperature increase. MR imaging provides a non-invasive temperature measurement, thermal dose quantification in the target tissue, utilizing the proton-resonance frequency (PRF) shift phenomena to monitor the temperature [13], and continuous imaging of the fibroid and surrounding structures such myometrium, bowel, and sacral nerves.

Previous studies have shown that MR-HIFU is capable of reducing the fibroid size and fibroid-related symptoms while maintaining an excellent safety profile [14–29]. In these studies, ablation of fibroid volumes is either done by the conventional approach, point-by-point technique, or by a volumetric heating technique. The first one is performed by iterative sonication of a single focal point, with each sonication followed by a cooling period. However, with this approach, a relatively large portion of the delivered energy is lost via diffusion of heat out of the small targeted region, and long treatment times are required. On the other hand, a volumetric heating technique where the focus of the ultrasound beam is electronically steered along a trajectory comprising of multiple outward-moving concentric circles with an axial diameter of 4–16 mm has been introduced [22, 30, 31].

The aim of this study was to evaluate the safety and effectiveness of MR-HIFU therapy using a volumetric ablation technique in the treatment of symptomatic uterine fibroids in China.

## Methods

This study was a multi-center, single arm, non-randomized clinical trial to evaluate the safety and effectiveness of volumetric MR-HIFU system in the treatment of symptomatic uterine fibroid patients (sponsored by Philips, clinicaltrials.gov identifier NCT01588899). Local ethics committee approval was obtained from both Peking University First Hospital and the First Affiliated Hospital, Xi’an Jiaotong University for the protocol prior to study initiation. Written informed consent was obtained from each patient at the screening visit prior to the initiation of any study-related procedures.

A total of 350 patients were screened for the trial, out of which 107 were included. Inclusion criteria were as follows: (1) women aged >18 years; (2) weight <140 kg; (3) pre- or peri-menopausal; (4) MR-HIFU device accessibility to treat at least 50 % of the total fibroid volume; (5) total planned ablation volume of all fibroids should not exceed 250 ml; (6) dominant fibroid (diameter) is greater than or equal to 3 cm; (7) no highly perfused or brighter than myometrium in T2-weighted MRI (a.k.a. type 3 per Funaki classification [32]); and (8) willing and able to attend all study visits. Exclusion criteria were as follows: (1) other pelvic disease; (2) desire for future pregnancy; (3) positive pregnancy test; (4) hematocrit <25 %; (5) surgical clips in the direct path of the HIFU beam; and (6) MRI contrast agent contraindicated.

**Fig. 1:**
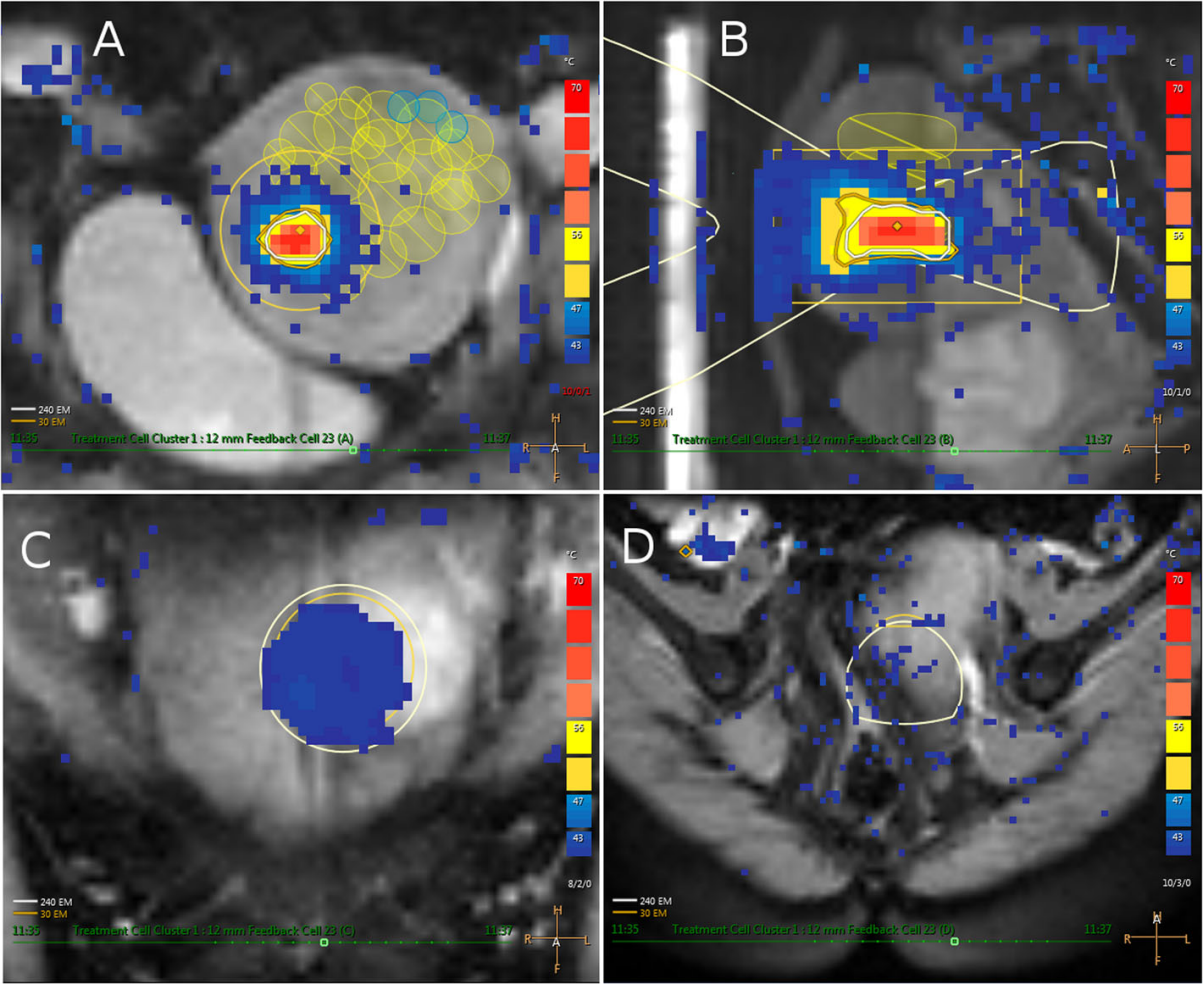
Multiplane MR thermometric images acquired during sonication with a 12-mm treatment cell (frequency, 1.2 MHz; acoustic power, 190 W) with visualization of the ultrasound focus in **a** coronal view, **b** sagittal view, and **c** coronal view from the near field and **d** oblique view from the far field

All therapies were conducted using a clinical MR-HIFU system (Sonalleve V2, Philips, Best, the Netherlands) integrated into a 1.5T MR scanner at Xi’an site and 3.0T MR at Peking site (Achieva, Philips, Best, the Netherlands). The details of the MR-HIFU system and treatment procedures have been described elsewhere [16]. T2-weighted (T2W) 3D turbo spin-echo (TSE) images were acquired for screening and treatment planning. RF-spoiled gradient-recalled EPI sequence used for real-time thermometry had three coronal slices perpendicular to the beam axis, centered at the focal-region and one sagittal slice aligned along the beam propagation direction and two additional slices were positioned to monitor potential excessive near field (i.e., on the skin) and far-field (Fig. 1a–d). Immediately after HIFU treatment and at follow-ups, a contrast-enhanced T1-weighted (CE-T1W) image was acquired.

Patient immobilization time was limited to 3 h due to the risk of deep venous thrombosis. For analgesics, all patients received 200 mg Celebrex at Peking site and oxycodone hydrochloride and Paracetamol (acetaminophen) tablets (5 mg) at Xi’an site.

### Study endpoints

#### The primary endpoints

The primary endpoint of the study, clinical efficacy, was based on the proportion of treated patients with fibroid shrinkage as measured with MRI without additional treatment (myomectormy, hysterectomy, uterine artery embolization, or MR-HIFU) during the follow-up period [20, 25, 26]. An image-based primary endpoint was selected in order to be in line with the Chinese SFDA Notice 222 (2007): Notice on the issuance of the relevant technical requirements for HIFU device.

Women were defined as having fibroid shrinkage, i.e., treatment success at 6 months if the total volume of their treated fibroids plus measurement error estimate was 90 % or less then than the baseline fibroid volume minus measurement error estimate. Similarly, women were considered as treatment failures (no fibroid shrinkage) if the total volume of their treated fibroids at 6 months plus measurement error estimate was more than 90 % of the total baseline fibroid volume minus measurement error. The estimation of the measurement error was based on intra-reader variability and was calculated by the core laboratory responsible for image evaluation. Fibroid volume was estimated by manual segmentation on T2W MRI at baseline (treatment day, prior to treatment) and at 1, 3, and 6 months post treatment. Measurements were performed by an independent reader (CRO’s core lab) blinded to the patient information. All patients who underwent HIFU treatment were to be included in the primary efficacy analysis.

In addition to the primary endpoint expressed as a binary variable, the percent reduction in fibroid volume was also calculated using the following formula:

$$ \%\ \mathrm{Reduction}=\frac{\mathrm{Total}\ \mathrm{Volume}\ \mathrm{at}\ \mathrm{Baseline} - \mathrm{Total}\ \mathrm{Volume}\ \mathrm{at}\kern0.5em 6\ \mathrm{months}}{\mathrm{Total}\ \mathrm{Volume}\ \mathrm{at}\ \mathrm{Baseline}}\times 100 $$

#### The secondary endpoints

In this study, the secondary endpoints were chosen to match endpoints commonly used internationally in the field of fibroid follow-up.

- Evaluation of the non-perfused volume assessed by MRI: The non-perfused volume (NPV) was estimated using a manual contour segmentation on CE-T1W images immediately post treatment, 1, 3, and 6 months post treatment. Lesion NPV measurements were performed by an independent reader (CRO’s core lab) blinded to the patient information. The percentage of the fibroid volume that was non-perfused was calculated as follows:
  $$ \mathrm{N}\mathrm{P}\mathrm{V}\ \left(\%\right)=\frac{\mathrm{Non}\ \hbox{-} \mathrm{perfused}\ \mathrm{V}\mathrm{olume}}{\mathrm{Fibroid}\ \mathrm{V}\mathrm{olume}}\times 100 $$
- Evaluation of The Uterine Fibroid Symptom and Quality of Life (UFS-QoL) questionnaire with symptom severity scoring (SSS): UFS-QoL questionnaire was completed at screening, 1, 3, and 6 months post treatment. The UFS-QoL has seven subscales (i.e., SSS, concern, activities, energy/mood, control, self-conscious, and sexual function) and was validated in a study comparing scores between women with normal menstrual cycles and women with symptomatic fibroids [3]. The SSS (questions 1 to 8) ranges from 8 to 40 (i.e., range = 32). Higher transformed SSS values are indicative of greater symptom severity or bother and lower scores indicate minimal symptom severity. The HRQL has six subscales (i.e., concern, activities, energy/mood, control, self-conscious, and sexual function). Each subscale was created by summing the scores of the items. Higher transformed HRQL score is an indicative of better quality of life. The transformed SSS score and transformed HRQL used in this study analysis were calculated as described by Spies et al. [3].

### The safety endpoints

Adverse events (AEs) were based on patient self-report and collected by recording the self-reported symptoms at site visits and during telephone contacts. AEs including serious adverse events (SAEs) were reviewed by the sponsor and the clinical events committee (CEC). SAEs were defined according to the Society of Interventional Radiology guidelines [33].

### Statistical analysis

The primary analysis tested the null hypothesis, which the proportion of treated women with fibroid shrinkage would be equal to 0.70, against the alternate hypothesis, which the proportion with fibroid shrinkage would be greater than 0.70. This hypothesis was evaluated using data for both sites and a two-sided exact-binomial *Z*-test at the 0.05 significance level. Exact Clopper-Pearson 95 % confidence interval for the proportion of patients with fibroid shrinkage at 6 months was calculated.

In addition, the primary analysis was repeated with fibroid shrinkage and was defined more conservatively, that is the proportions of patients with ≥10, ≥20, ≥30, ≥40, and ≥50 % reduction in fibroid volume at 6 months was estimated, with the Exact Clopper-Pearson 95 % confidence interval for the proportions calculated overall and by site. The proportion of patients with fibroid shrinkage 1, 3, and 6 months post treatment with 95 % CI was also calculated. Also, the percent reduction in fibroid volume at 1, 3, and 6 months follow-up were calculated based on fibroid volume measured using MRI. The NPV based on MRI was summarized by site and overall.

The transformed SSS and seven UFS-QoL subscales were summarized for baseline, 1, 3, and 6 months post treatment. The number of observations, mean, standard deviation, and 95 % CI for the raw values and for change from baseline were presented by site and overall.

Statistical analyses were performed using SAS (ver. 9.3, SAS Institute Inc., Cary, NC 27513, USA).

## Results

### Patient demographics

**Fig. 2:**
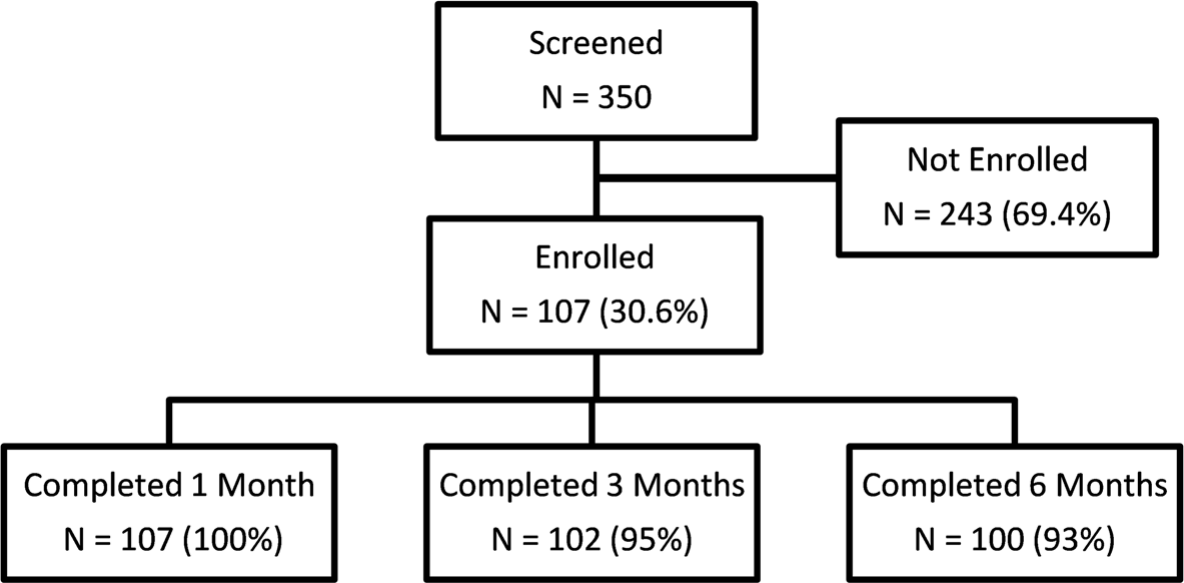
Flowchart of patient screening and enrollment

Three hundred fifty women were screened, and 107 (30.6 %) of them underwent HIFU treatment. A summary of patient screening is shown in Fig. 2. Findings from the MRI screening which includes presence of vertical scar tissue, bowel interference, patients with more than five symptomatic fibroids, fibroids that are too large (>250 cm^3^) or too deep (skin to fibroid center >10 cm), highly perfused fibroid with excessive vascularity (Funaki type 3), pedunculated fibroids were the most frequent reason (i.e., 69.5 %) for a patient not to be enrolled. Also, 29.6 % of the screened women withdrew from study prior to enrollment. All of the 107 treated patients completed the 1-month follow-up visit, 102 (95 %) completed the 3-month visit, and 100 (93 %) completed the 6-month visit. A summary of patient demographics is presented in Table 1. No MR-HIFU therapies were canceled due to technical failures.

**Table 1:** Patient demographics, number and type of fibroids

Variable	Category	Peking (*N* = 58)	Xi’an (*N* = 49)	Overall (*N* = 107)
Age (years)	Mean (SD)	45.1 (4.50)	43.9 (4.50)	44.5 (4.50)
Weight (kg)	Mean (SD)	62.2 (7.60)	58.5 (7.80)	60.5 (7.90)
BMI (kg/m^2^)	Mean (SD)	24 (2.40)	22.8 (2.60)	23.5 (2.60)
Ethnicity	Asian	57 (98.3 %)	49 (100 %)	106 (99.1 %)
	Caucasian	1 (1.7 %)	0 (0.0 %)	1 (0.9 %)
Number of fibroids	Total number	77	53	130
	Mean (SD)	1.3 (0.50)	1.1 (0.30)	1.2 (0.50)
Type of fibroid	Intramural	42 (54.5 %)	45 (84.9 %)	87 (66.9 %)
	Submucosal	5 (6.5 %)	2 (3.8 %)	7 (5.4 %)
	Subserosal	30 (39.0 %)	6 (11.3 %)	27.7 %)

### Primary efficacy results

#### Reduction in fibroid volume: patient level analysis

The overall proportion of women with treatment success (the total fibroid volume of treated fibroids at 6 months plus the error estimate was 90 % or less than the total fibroid volume of treated fibroids at baseline minus the error estimate) was 0.93 (95 % CI 0.86–0.97). The error estimate was based on intra-reader variability calculated by the core laboratory responsible for the image evaluation. The proportion of treatment success is significantly higher than the null hypothesized value of 0.70 for overall and for each site, *p* value <0.001. Treatment success was reported in 52 of the 58 (90 %) patients treated at the Peking site and 47 of the 49 (96 %) patients treated in Xi’an Jiaotong site. Table 2 shows the number and proportion of patients with fibroid shrinkage by study site and overall.

**Table 2:** Patients with fibroid shrinkage at 6 months post treatment

Sites	Evaluable patients	Number of successes	Proportion	95 % CI	*P* value
Peking	58	52	0.90	(0.79, 0.96)	<.001
Xi’an	49	47	0.96	(0.86, 1.00)	<.001
Overall	107	99	0.93	(0.86, 0.97)	<.001

#### Reduction in fibroid volume: fibroid level analysis

The proportion of patients with ≥10, ≥20, ≥30, ≥40, and ≥50 % reduction in fibroid volume 6 months post treatment with the Exact Clopper-Pearson 95 % confidence interval overall and by site are presented in Table 3. The proportion of patients with at least 40 % reduction in total fibroid volume was 0.61 (95 % CI 0.51–0.71).

**Table 3:** Proportion of patients with reduction in total fibroid volume at 6 months

Reduction in total fibroid volume (%)	Peking (*N* = 58)	Xi’an (*N* = 49)	Overall (*N* = 107)
≥10	0.90 (0.79, 0.96)	0.96 (0.86, 1.00)	0.93 (0.86, 0.97)
≥20	0.89 (0.77, 0.96)	0.87 (0.74, 0.95)	0.88 (0.80, 0.94)
≥30	0.76 (0.62, 0.87)	0.74 (0.59, 0.86)	0.75 (0.65, 0.83)
≥40	0.69 (0.54, 0.80)	0.52 (0.37, 0.67)	0.61 (0.51, 0.71)
≥50	0.46 (0.33, 0.60)	0.30 (0.18, 0.46)	0.39 (0.29, 0.49)

### Secondary efficacy analysis

#### Fibroid-related endpoints

**Fig. 3:**
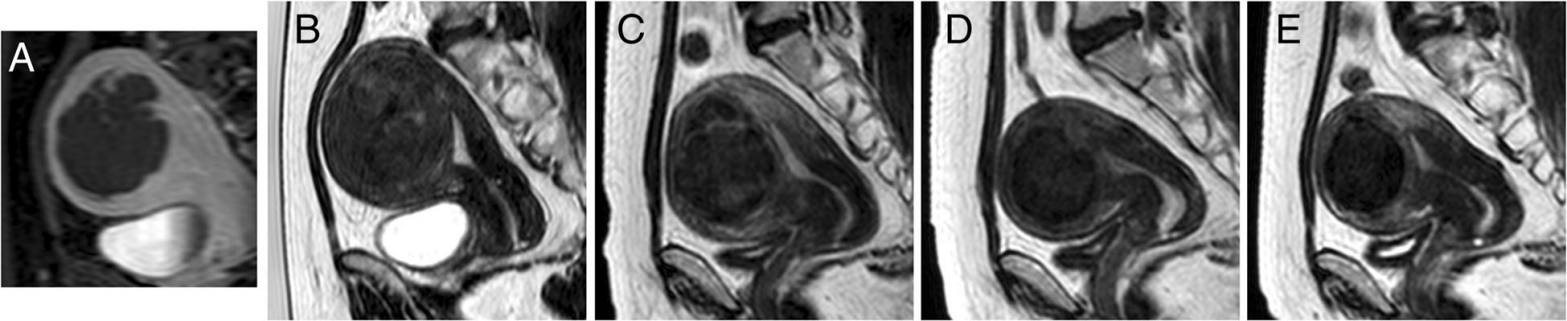
An example of the progression of fibroid volume reduction after successful treatment: **a** T1W CE-THRIVE immediately post treatment, **b** T2W image of the fibroid at treatment day (fibroid volume 144.3 ml), and **c**–**e** T2W images at 1 month (70.3 ml, % reduction 51.32 %), 3 months (31.8 ml, % reduction 74.8 %), and 6 months (23.6 ml, % reduction 83.6 %) follow-ups

The percent reduction in fibroid volume at 1, 3, and 6 months follow-up based on fibroid volume measured using MRI both in Peking and Xi’an sites were very consistent between the two sites (20.6, 43.3, and 51.3 % for Peking site and 20.1, 39.6, and 48.85 % for Xi’an). The number of observations, mean, standard deviation, and 95 % CI is presented in Table 4. Overall, the mean percent reduction in fibroid volume at 6 months was 50.2 %, with the lower bound of the 95 % CI of 46.1. This result indicates that on the average, the fibroid volume reduction for this procedure is significantly greater than 45 %. Figure 3 shows an example of the progression of fibroid volume reduction after successful treatment.

**Table 4:** Percent reduction in fibroid volume measured by MRI

		Months post treatment		
Site	Parameter	1	3	6
Peking	No. patients	58	54	54
	Mean (SD)	20.6 (23.00)	43.3 (25.10)	51.3 (21.60)
	95 % CI	(14.6, 26.6)	(36.4, 50.1)	(45.4, 57.2)
Xi’an	No. patients	49	45	46
	Mean (SD)	20.1 (16.60)	39.6 (19.40)	48.8 (19.00)
	95 % CI	(15.3, 24.9)	(33.8, 45.4)	(43.2, 54.5)
Overall	No. patients	107	99	100
	Mean (SD)	20.4 (20.20)	41.6 (22.70)	50.2 (20.40)
	95 % CI	(16.5, 24.2)	(37.1, 46.1)	(46.1, 54.2)

Table 5 provides a summary on NPV after treatment. The mean NPV was also very consistent between the two sites (i.e., 55.7 % for the Peking site and 53.7 % for the Xi’an site).

**Table 5:** Summary non-perfused fibroid volume percent (%NPV) based on MRI evaluation (treatment visit)

Variable	Category	Peking (*N* = 58)	Xi’an (*N* = 49)	Overall (*N* = 107)
%NPV	No. patients	57	49	106
	Mean (SD)	55.7 (21.10)	53.7 (21.30)	54.8 (21.20)
	95 % CI	(50.1, 61.3)	(47.5, 59.8)	(50.7, 58.8)

*%NPV* percent non-perfused volume

#### Uterine fibroid symptom quality of life (UFS-QoL)

The UFS-QoL was assessed at the screening visit and during follow-up at 1, 3, and 6 months. The overall results of UFS-QoL are shown in Table 6. The SSS-transformed score for both sites combined were observed to decline over time. The SSS-transformed score at screening was 34.4, and 24.0 at 6 months post treatment. The SSS at 6 months is statistically different from the screening SSS at 0.05 level (non-overlapping 95 % CI). At the same time, the HRQL-transformed score increased from 73 to 82.2. The HRQL at 6 months is statistically different from the screening HRQL at 0.05 level (non-overlapping 95 % CI).

**Table 6:** Uterine fibroid symptom severity (UFS-QoL) overall summary

			Months post treatment		
UFS-QoL parameter	Statistic	Screening (*N* = 107)	1 (*N* = 107)	3 (*N* = 102)	6 (*N* = 100)
SSS transformed^a^					
	Mean (SD)	34.4 (14.70)	28.1 (15.00)	27.2 (15.70)	24 (16.10)
	95 % CI	(31.6, 37.2)	(25.2, 30.9)	(24.2, 30.3)	(20.8, 27.2)
HRQL transformed^a^					
	Mean (SD)	73 (18.80)	78.1 (18.00)	78.5 (19.50)	82.2 (17.30)
	95 % CI	(69.4, 76.6)	(74.7, 81.6)	(74.7, 82.4)	(78.7, 85.6)
Total score					
	Mean (SD)	79.3 (25.10)	71.3 (24.40)	70.6 (26.10)	65.4 (24.30)
	95 % CI	(74.5, 84.1)	(66.7, 76)	(65.5, 75.7)	(60.5, 70.2)

*SSS* symptom severity score, *HRQL* health-related quality of life

^a^Transformed scores (SSS and HRQL) can range from 0 to 100. The total score ranges from 37 to a maximum of 185

### Adverse events

Table 7 gives an overview of AEs. A total of 366 adverse events were reported, out of which 10 were reported as SAEs. No deaths were reported. A large number of SAEs is explained by four pregnancies, each of which was reported as SAE when they occurred. All pregnant patients terminated their pregnancy voluntarily while enrolled in the trial. Each pregnancy termination was reported as a separate SAE as well. The remaining two SAEs were due to occurrence of breast cancer surgery (*N* = 1) and fibroid-related minimally invasive surgery (*N* = 1). All SAEs were considered unrelated to study procedure and device.

**Table 7:** Summary of adverse events

Adverse event	Peking (*N* = 58)	Xi’an (*N* = 49)	Overall (*N* = 107)
Total number of adverse events	224	142	366
Patients with at least one SAE	1 (1.7 %)	5 (10.2 %)	6 (5.6 %)
Total number of serious AEs	2 (0.9 %)	8 (5.7 %)	10 (2.7 %)
Pregnancy	1 (1.7 %)	3 (6.1 %)	4 (3.7 %)
Termination of pregnancy	1 (1.7 %)	3 (6.1 %)	4 (3.7 %)
Ductal carcinoma	0 (0.0 %)	1 (2.0 %)	1 (0.9 %)
Fibroid surgery	0 (0.0 %)	1 (2.0 %)	1 (0.9 %)
Patients with at least one AE	45 (77.6 %)	38 (77.6 %)	83 (77.6 %)
Total number of related AEs	52 (23.2 %)	107 (74.8 %)	159 (43.3 %)
Abdominal/pelvic pain	30 (51.7 %)	30 (61.2 %)	60 (56.1 %)
Skin heating/pain	25 (43.1 %)	10 (20.4 %)	35 (32.7 %)
Leg pain	15 (25.9 %)	5 (10.2 %)	20 (18.7 %)
Back pain	11 (19.0 %)	2 (4.1 %)	13 (12.1 %)
Abdominal distension	3 (5.2 %)	1 (2.0 %)	4 (3.7 %)
Buttock pain	4 (6.9 %)	0 (0.0 %)	4 (3.7 %)
Leg numbness	3 (5.2 %)	0 (0.0 %)	3 (2.8 %)
Skin redness	1 (1.7 %)	2 (4.1 %)	3 (2.8 %)
1st degree skin burn	1 (1.7 %)	0 (0.0 %)	1 (0.9 %)
Leg edema/pain	1 (1.7 %)	0 (0.0 %)	1 (0.9 %)
Pubic bone pain	0 (0.0 %)	1 (2.0 %)	1 (0.9 %)
Skin edema	1 (1.7 %)	0 (0.0 %)	1 (0.9 %)

Out of the non-serious AEs, 159 (43.7 %) were considered to be related or possibly related to the device. Most patients (i.e., 77.6 %) reported at least one AE related to the device, most commonly abdominal/pelvic pain, skin heating/pain, leg pain, and back pain. Of 159 AEs, 153 were recorded during and immediately after HIFU treatment while six of them were 1–3 days after treatment.

## Discussion

In this study, the effectiveness of MR-HIFU device using a volumetric ablation technique in the treatment of symptomatic uterine fibroids was measured by assessing shrinkage of treated fibroids, which is an objective, quantitative, imaging-based study endpoint.

The study met its pre-defined endpoint, with 93 % (95 % CI 86–97 %) of study patients exhibiting shrinkage of ≥10 % fibroid volume shrinkage. In this study, the mean fibroid volume reduction at 6 months was 50.3 % (Table 4). As shown in Table 5, this rate of success was achieved with a mean NPV of 54.8 %. NPV as a predictor for fibroid shrinkage and clinical success has been established in one of the latest studies [24]. Given the adequate level of NPV achieved in this study, the fibroid volume is reduced as expected. Note that NPV is expected to further improve as the operators complete their learning curve and gain more experience in patient selection and conducting the procedure [34, 35].

Patients’ SSS of the UFS-QoL questionnaire improved after treatment. As shown in Table 6, the mean SSS was reduced from 34 points at baseline to 24 points at 6 months post treatment. A reduction of 10 points or more is considered clinically significant [16]. It must be noted that, in the present population in China, patients scored their own symptoms considerably lower, and their personal health better, than in predicate studies. Patients with similar demographics in Western population typically scored a baseline of over 60 points [24] compared to 34 points in a Chinese population. This difference can most likely be attributed to cultural differences in self-perception. Evidently, a mean SSS of around 30 points was sufficient for the patients to seek treatment for their fibroid symptoms in this study. However, the results of this prospective study demonstrate that the relationship between volume reduction and symptomatic improvement in fibroids following MR-HIFU treatment is not a direct correlation.

In this study, AEs reported were predominantly mild. Ten SAEs were reported, all of which were judged to be unrelated to the treatment procedure. Reported AEs typically relate to heating from the ultrasound beam, and are typically manifested inter-procedurally and resolved shortly thereafter. This is in line with previous clinical studies on MR-HIFU treatments of uterine fibroids [18, 23]. In one patient, buttock/leg/foot pain persisted for 44 days following the HIFU treatment; isolated similar events have been observed previously in studies using other MR-HIFU devices [16, 36]. An independent CEC evaluated the reported adverse events for determination of seriousness and causal relationship with the treatment. The CEC concluded that most of the AEs were minor, and the safety profile of the MR-HIFU system was acceptable.

A limitation of the study was the single-arm design. MR-HIFU treatment was not directly compared to other options for uterine fibroid therapy. Prior experience shows however that randomization can be prohibitive for enrollment when one arm is invasive and the other non-invasive. To limit possible bias, an objective measurement was chosen as primary endpoint for the study, and assessed by an independent CRO core laboratory. The choice of an objective, a quantitative endpoint which can be independently measured, adds strength to the study. It should be noted however that the secondary endpoints provide symptomatic follow-up and present as important a result as the primary image-based endpoint.

## Conclusions

This study demonstrated that MR-HIFU device using a volumetric ablation technique is safe and technically effective and can be utilized in clinically efficient treatments of symptomatic uterine fibroids.

## Abbreviations

AE: Adverse events
CEC: Clinical events committee
CE-T1W: Contrast-enhanced T1-weighted
HIFU: High-intensity focused ultrasound
MR-HIFU: Magnetic resonance-guided high-intensity focused ultrasound
NPV: Non-perfused volume
PRF: Proton resonance frequency
SAE: Severe adverse events
SSS: Symptom severity score
T2W-TSE: T2-weighted turbo spin echo
UAE: Uterine artery embolization
UFS-QoL: Uterine fibroid symptom quality of life
US: Ultrasound
